# Reduced heart rate variability predicts incident diabetic polyneuropathy

**DOI:** 10.3389/fendo.2026.1880532

**Published:** 2026-07-16

**Authors:** Dimitrios Tsilingiris, Daniel Schmalzridt, Omar Eldesouky, Florian Kalb, Viktoria Flegka, Ekaterina von Rauchhaupt, Theresa Hoefer, Stefan Kopf, Thomas Fleming, Stephan Herzig, Anna Hohneck, Julia Szendroedi, Zoltan Kender

**Affiliations:** 1 Department of Endocrinology, Diabetology, Metabolic Diseases and Clinical Chemistry (Internal Medicine I), University Hospital Heidelberg, Heidelberg, Germany; 2 First Department of Internal Medicine, University Hospital of Alexandroupolis, Demokritus University of Thrace, Alexandroupolis, Greece; 3 German Center for Diabetes Research (DZD), Munich-Neuherberg, Germany; 4 Department of Internal Medicine 1, Endocrinology & Diabetes, University of Lübeck, Lübeck, Germany; 5 Joint Heidelberg-Institute for Diabetes and Cancer (IDC) Translational Diabetes Program, Internal Medicine I, Heidelberg University Hospital, Heidelberg, Germany; 6 Institute for Diabetes and Cancer, Helmholtz Center Munich, Neuherberg, Germany

**Keywords:** cardiovascular autonomic neuropathy, diabetes, diabetic neuropathy, distal sensorimotor polyneuropathy, heart rate variability, quantitative sensory testing

## Abstract

**Objective:**

To determine whether cardiovascular autonomic neuropathy (CAN) and reduced heart rate variability (HRV) predict the development of distal symmetrical polyneuropathy (DSPN) in individuals with diabetes mellitus (DM).

**Methods:**

A total of 288 individuals with DM (mean age 58.1 ± 13.4 years, 41.3% women, 78.5% with type 2 diabetes) underwent baseline assessment of CAN using cardiovascular autonomic reflex tests and HRV indices (CVRR, rMSSD, high- and low-frequency power). DSPN was characterized using three definitions: (i) clinical criteria based on symptoms and signs, (ii) the sensory-loss phenotype derived from quantitative sensory testing, and (iii) the Toronto consensus definition requiring abnormal nerve conduction plus symptoms or signs. A total of 194 participants without DSPN at baseline were followed for 3.0 ± 1.3 years to assess incident DSPN.

**Results:**

At baseline, CAN prevalence was 16.3%, and low HRV indices were present in 18.6–40.2% of participants. DSPN prevalence was 27.7%, 17.6%, and 20.8% for definitions (i), (ii), and (iii), respectively. Cross-sectionally, CAN and reduced HRV were associated with DSPN (unadjusted ORs 1.8–4.0), with associations largely persisting after adjustment. Incident DSPN occurred at 8.4, 5.7, and 3.3 per 100 person-years. Baseline CAN independently predicted incident DSPN (adjusted HRs 6.20, 3.30, and 7.33 across definitions). Lower rMSSD, HF power, and LF power predicted incident DSPN according to definitions (i) and (iii), whereas reduced CVRR predicted DSPN defined by criteria (ii).

**Conclusions:**

Established CAN is a strong predictor of future DSPN. Reduced HRV provides a practical, accessible alternative marker for identifying individuals at elevated neuropathy risk.

## Introduction

Distal symmetrical polyneuropathy (DSPN) is a common microvascular complication of both type 1 and type 2 diabetes mellitus (DM), affecting up to half of individuals with diabetes during their lifetime (1). The associated loss of protective sensation is a leading cause of diabetic foot complications, including chronic ulceration and amputation, with major consequences for mobility, quality of life, and overall prognosis (2). Since no specific therapy exists to reverse DSPN, early identification of individuals at high risk is essential. However, commonly used screening tools lack sufficient sensitivity for early or preclinical disease, while more accurate methods—such as electrophysiology and quantitative sensory testing (QST)—are typically limited to specialized or research settings (3). This highlights the need for complementary approaches capable of identifying individuals at elevated risk before clinically overt neuropathy develops.

Cardiovascular autonomic neuropathy (CAN) is another manifestation of diabetic neuropathy and has been consistently associated with adverse outcomes in DM (4), including a proposed role in the development of diabetic foot complications (5). Numerous cross-sectional studies have shown a high degree of coexistence between CAN and DSPN across both type 1 and type 2 diabetes (6–13). This overlap is unsurprising, as both conditions emerge from peripheral nerve fibre damage and share several established risk factors, including older age, poor glycaemic control, adiposity, longer diabetes duration, and increased urinary albumin excretion (14, 15). Nonetheless, it remains unclear whether CAN precedes and predicts the later development of DSPN, since all existing evidence has been cross-sectional.

CAN also overlaps closely with reduced heart rate variability (HRV), a widely available ECG-derived measure of autonomic function (4, 15). HRV parameters have been used as surrogate markers for CAN in previous work (16), and both time-domain and frequency-domain indices are known to associate with autonomic dysfunction (17, 18). However, evidence linking HRV to DSPN is limited to small, cross-sectional studies using heterogeneous neuropathy definitions and incomplete HRV characterisation (19–21). Prospective data evaluating whether reduced HRV predicts incident DSPN are entirely lacking.

The aim of the present study was to evaluate whether the presence of CAN is associated with an increased risk of DSPN, both cross-sectionally and prospectively among individuals without DSPN at baseline. A further objective was to determine whether HRV metrics—derived from short-term ECG recordings—can serve as practical alternatives to formal CAN assessment for DSPN risk stratification. The analyses were conducted in a deeply phenotyped cohort of individuals with diabetes, allowing assessment of the predictive value of autonomic dysfunction across complementary measures of somatic nerve involvement.

## Materials and methods

### Study sample

Data were derived from the Heidelberg Study of Diabetes and Complications (HEIST-DiC) (22), conducted in accordance with the Declaration of Helsinki. All participants provided written informed consent, and the study protocol was approved by the Ethics Committee of Heidelberg University Hospital. The study is registered at ClinicalTrials.gov (NCT03022721). The cross-sectional analyses included all individuals with type 1 or type 2 diabetes who had baseline data available for cardiovascular autonomic reflex tests (CARTs), heart rate variability (HRV), and at least one DSPN definition (n = 279). The longitudinal analyses included participants without DSPN at baseline according to any of the three DSPN definitions and who attended at least one follow-up visit (total n = 194; n = 151, 181, and 166 for DSPN definitions (i), (ii), and (iii), respectively).

### Study procedures

The study protocol has been previously described (22). All examinations took place in the morning following an overnight fast of 8–12 hours. Study visits were rescheduled if participants had acute illness or experienced hypoglycaemia (<70 mg/dL) within the preceding 24 hours. Participants were instructed to abstain from smoking and to withhold antihypertensive medications and beta-blockers until completion of the examinations. Medical history and medication use were collected using standardized questionnaires. Approximately 20 mL of venous blood was drawn for standard laboratory assessments. CARTs and DSPN diagnostics were performed in the fasting state.

### Cardiovascular autonomic nerve function and heart rate variability assessment

CARTs and HRV measurements were obtained using a standard 3-lead ECG and analysed with the SUESS SUEmpathy^®^ 100 system (SUESS Medizin-Technik GmbH, Aue, Germany; software version SUE1-4.0). All participants refrained from beta-blocker and antihypertensive therapy for 24 hours prior to testing. Nevertheless, beta-blocker use was included as a covariate in multivariable models to account for any residual effects on HRV, particularly from agents with a longer half-life. CARTs included (i) Heart rate response to deep breathing, expressed as the expiratory-to-inspiratory (E/I) ratio, (ii) Heart rate response to standing, expressed as the 30:15 ratio (longest-to-shortest RR interval during posture change), and (iii) Postural blood pressure response (ΔSBP, ΔDBP) measured over 3 minutes after standing. CAN was defined as the presence of at least two pathological CARTs. Age-specific cut-offs for the 30:15 and E/I ratios were provided by the manufacturer. Postural hypotension was defined as a decrease in systolic BP ≥20 mmHg or diastolic BP ≥10 mmHg. HRV was measured during 5 minutes of spontaneous breathing, following international recommendations (23). Time-domain indices included the coefficient of variation of R–R intervals (CVRR) and the root mean square of successive differences (rMSSD). Frequency-domain indices included low-frequency (LF, 0.04–0.15 Hz, ms²) and high-frequency (HF, 0.15–0.50 Hz, ms²) power. Time-domain measures were available for all participants, while frequency-domain measures were available in 267 individuals (92.7%).

### Definitions of DSPN

To reflect the heterogeneity of DSPN diagnostics in clinical and research practice, DSPN status was determined using three complementary definitions. The first definition relied on clinical criteria based on the Neuropathy Symptom Score (NSS) and the Neuropathy Disability Score (NDS). DSPN was diagnosed when participants exhibited either moderate signs (NDS ≥6), irrespective of symptoms, or moderate neuropathic symptoms (NSS ≥5) in combination with mild signs (NDS 3–5), aligning with approaches commonly used in routine clinical care (24, 25). The second definition was based on the sensory-loss (SL) phenotype derived from quantitative sensory testing (QST). This approach applied the DFNS protocol and the Vollert et al. algorithm, which classifies sensory phenotypes using z-scores across 13 distinct QST domains. The SL phenotype on the right foot was taken to indicate DSPN (26). The third definition followed a modified version of Toronto consensus criteria for confirmed DSPN. This required evidence of large-fibre dysfunction on nerve conduction studies, defined as a sural sensory nerve action potential or conduction velocity below the 1st percentile together with at least one abnormal motor nerve parameter—such as peroneal or tibial conduction velocity or compound motor action potential below the 2.5th percentile. The more stringent threshold for sensory sural nerve parameters was chosen to increase specificity. The percentile cut-offs were derived from HEIST-DiC participants with normoglycemia and no evidence of neuropathy (see also, **Supplementary Material**). In addition, participants were required to have neuropathic symptoms or signs (NSS and/or NDS ≥3) to meet the diagnostic threshold (27). DSPN status was available in 285 (99.0%), 273 (94.8%), and 269 (93.4%) individuals according to definitions (i), (ii), and (iii), respectively. For longitudinal analyses, only participants without DSPN at baseline were included, resulting in 151, 181, and 166 individuals for definitions (i), (ii), and (iii), respectively.

### Statistical analysis

Analyses were performed using IBM SPSS Statistics (version 25.0) and GraphPad Prism (version 9.4.1). Continuous variables are reported as mean (SD) or median (IQR), and categorical variables as n (%). Group comparisons used Student’s t-test or Mann–Whitney U test for continuous variables and chi-squared test for categorical variables. ROC curve analysis and Youden’s J index were used to identify optimal HRV cut-offs as surrogates for CAN. Cross-sectional associations between predictors (CAN, HRV) and DSPN were assessed with binary logistic regression. Longitudinal associations with incident DSPN were analysed using Cox proportional hazards models. Multivariable logistic and Cox models included adjustments for age, sex, BMI, waist-to-hip ratio (WHR), beta-blocker therapy, urinary albumin-to-creatinine ratio (ACR), diabetes duration, HbA1c, and estimated glomerular filtration rate (eGFR, CKD-EPI). Odds ratios (ORs) and hazard ratios (HRs) with 95% confidence intervals (CIs) are reported. A p-value <0.05 was considered statistically significant.

## Results

### Characteristics of the studied population

A total of 288 individuals with diabetes (226 with type 2 diabetes, 78.5%) underwent baseline CAN testing and were included in the cross-sectional analyses. The prevalence of CAN was 16.3%. Among those without CAN (n = 241, 83.7%), 152 participants (63.1%) exhibited no abnormalities in CARTs, whereas 89 (36.9%) had one abnormal test. Among those with CAN (n = 47), nearly all individuals (93.6%) had two abnormal tests, and 6.4% had abnormalities in all three tests. Abnormalities were most commonly observed in the E/I ratio, while postural hypotension was uncommon (**Supplementary Table 1**). Individuals with CAN displayed higher BMI (31.6 vs. 29.4 kg/m²) and higher HbA1c (7.8% vs. 7.2%) than those without CAN. As expected, HRV indices were consistently lower among participants with CAN (**Table 1**).

**Table 1:** Baseline characteristics of participants with and without cardiovascular autonomic neuropathy (CAN). .

Parameter	No CAN (n=241)	CAN (n=47)	P-value
Age (years)	58.2 (13.7)	57.7 (12.0)	0.788
Sex (female)	102 (42.3%)	17 (36.2%)	0.441
Diabetes type (type 2)	186 (77.2%)	40 (85.1%)	0.226
Diabetes duration (years)	10.0 (4.0–18.0)	10.0 (5.0–19.0)	0.481
HbA1c (% [mmol/mol])	7.2 (1.2) [55 (13)]	7.8 (1.7) [62 (18)]	0.036
BMI (kg/m²)	29.4 (6.0)	31.6 (5.6)	0.022
Waist–hip ratio	0.96 (0.10)	0.98 (0.07)	0.201
Beta-blocker use	61 (27.4%)	13 (25.5%)	0.787
eGFR (ml/min/1.73 m²)	93.2 (17.3)	92.3 (20.6)	0.753
Urine ACR (mg/g)	8.6 (4.8–24.3)	13.0 (5.0–40.4)	0.074
DSPN (i)*	66/238 (27.5%)	20/47 (42.6%)	0.043
DSPN (ii)*	35/228 (15.5%)	13/45 (28.9%)	0.029
DSPN (iii)*	40/225 (18.1%)	16/44 (36.3%)	0.005
CVRR (%)	3.57 (1.75)	2.45 (1.29)	<0.001
rMSSD (ms)	23.5 (19.5)	10.9 (7.8)	<0.001
HF power (ms²)	253.8 (455.1)	61.0 (142.9)	<0.001
LF power (ms²)	514.9 (673.5)	191.0 (372.5)	<0.001

Values are mean (SD), median (IQR), or n (%), depending on distribution. *Denominators differ due to missing DSPN assessments.

### Relationship of HRV with CAN

ROC curve analysis identified narrow optimal cutoff ranges for each HRV index based on Youden’s J statistic: CVRR 2.0–2.2%, rMSSD 12–14 ms, HF power 45–50 ms², and LF power 100–120 ms² (**Supplementary Figure 1**). Logistic regression analyses demonstrated that values within these ranges were strongly predictive of baseline CAN, independent of covariates (**Supplementary Table 2**). Slight differences in optimal thresholds emerged when focusing on cross-sectional DSPN associations (2.0%, 12 ms, 45 ms², 100 ms² for CVRR, rMSSD, HF, and LF, respectively) versus longitudinal prediction of incident DSPN (2.2%, 14 ms, 50 ms², 120 ms²), suggesting differential associations of HRV with established versus future DSPN.

### Risk factors for CAN, low HRV and DSPN

Exploratory multivariable regression analysis identified several factors associated with CAN, low HRV, and DSPN (**Supplementary Table 3**). Both CAN and reduced HRV were generally linked to poorer glycemic control, reflected by higher HbA1c values (CAN: aOR 1.31, 95% CI 1.05–1.64; rMSSD: aOR 1.41, 95% CI 1.15–1.72; LF power: aOR 1.37, 95% CI 1.10–1.70). Measures of adiposity also demonstrated associations, including BMI with CAN (aOR 1.05, 95% CI 1.00–1.11) and WHR with HF and LF power (aOR 42.04 and 39.78, respectively). Older age was associated with reduced CVRR and LF power. Higher urinary albumin excretion (log ACR) predicted low rMSSD and showed similar trends for CVRR and HF power. Reduced rMSSD and HF power were also associated with male sex and longer diabetes duration.

For DSPN, older age consistently predicted its presence across all three definitions (aORs 1.4–1.7). Higher urinary albumin excretion was associated with both QST-defined and Toronto-defined DSPN (aORs 2.42 and 2.34, respectively) and showed a similar but nonsignificant trend for clinical DSPN (aOR 1.48). Male sex was independently associated with Toronto-defined DSPN (aOR 6.62).

### Cross sectional association between CAN/HRV and DSPN

**Figure 1:**
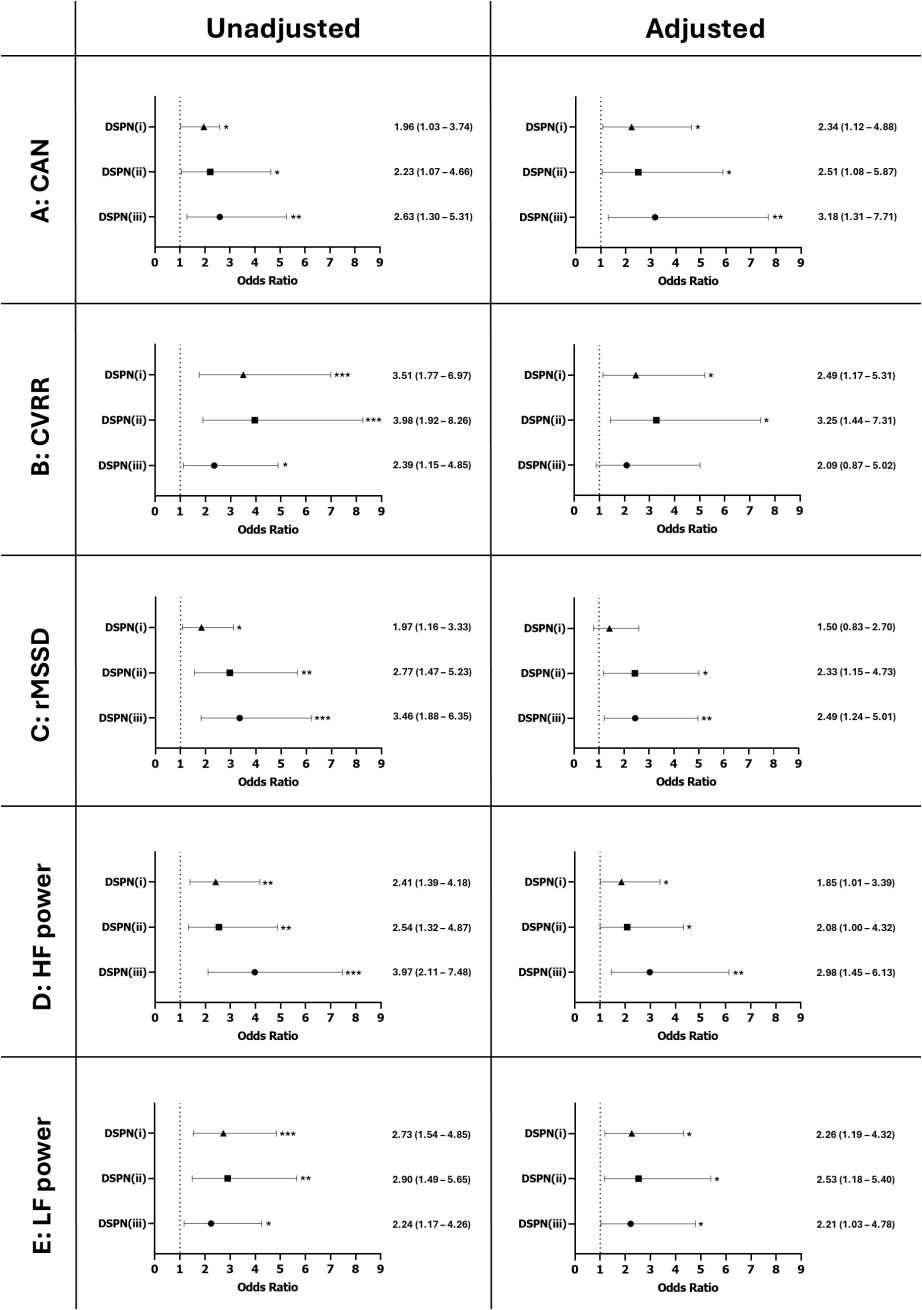
Cross-sectional associations of cardiovascular autonomic neuropathy (CAN) **(A)** and low heart rate variability (HRV) **(B–E)** with the presence of distal symmetrical polyneuropathy (DSPN) at baseline. *Left panels* display crude odds ratios (ORs) with 95% confidence intervals; *right panels* show ORs adjusted for age, sex, diabetes duration, body mass index, waist-to-hip ratio, beta-blocker use, estimated glomerular filtration rate (eGFR), HbA1c, and urine albumin-to-creatinine ratio (uACR). Abbreviations: CAN, cardiovascular autonomic neuropathy; CVRR, coefficient of variation of R–R intervals; rMSSD, root mean square of successive differences; HF power, high-frequency power; LF power, low-frequency power. *, **, *** indicate p <0.05, p <0.01, and p <0.001, respectively.

DSPN prevalence was higher among participants with CAN than among those without CAN across all definitions: 42.6% vs. 27.7% for DSPN (i), 28.9% vs. 15.4% for DSPN (ii), and 36.3% vs. 17.8% for DSPN (iii) (all p < 0.05). Multivariable logistic regression confirmed that CAN was independently associated with DSPN irrespective of the definition used (**Figure 1**).

Low HRV also demonstrated consistent cross-sectional associations with DSPN. Using the HRV cutoffs predictive of CAN, both HF and LF power were independently associated with DSPN across all definitions. Associations for CVRR and rMSSD remained significant in most models, although the relationship between CVRR and DSPN (iii) and between rMSSD and DSPN (i) attenuated after multivariable adjustment. Overall, low HRV conferred approximately 1.8- to 4-fold higher odds of DSPN in univariable models and 1.5- to 3-fold higher odds in adjusted models (**Figure 1**).

### Longitudinal analysis

A total of 194 participants without DSPN at baseline were included in the longitudinal analysis (n = 151, 181, and 166 for DSPN definitions (i), (ii), and (iii), respectively). At study entry, CAN was present in 11.3% of this subgroup, while low CVRR, rMSSD, HF power, and LF power were observed in 18.6%, 40.2%, 27.8%, and 26.3% of participants, respectively. Over a mean follow-up period of 3.0 ± 1.3 years, incident DSPN occurred in 37 (25.2%), 30 (17.0%), and 16 (9.9%) individuals according to definitions (i), (ii), and (iii), corresponding to incidence rates of 8.4, 5.7, and 3.3 per 100 person-years.

**Figure 2:**
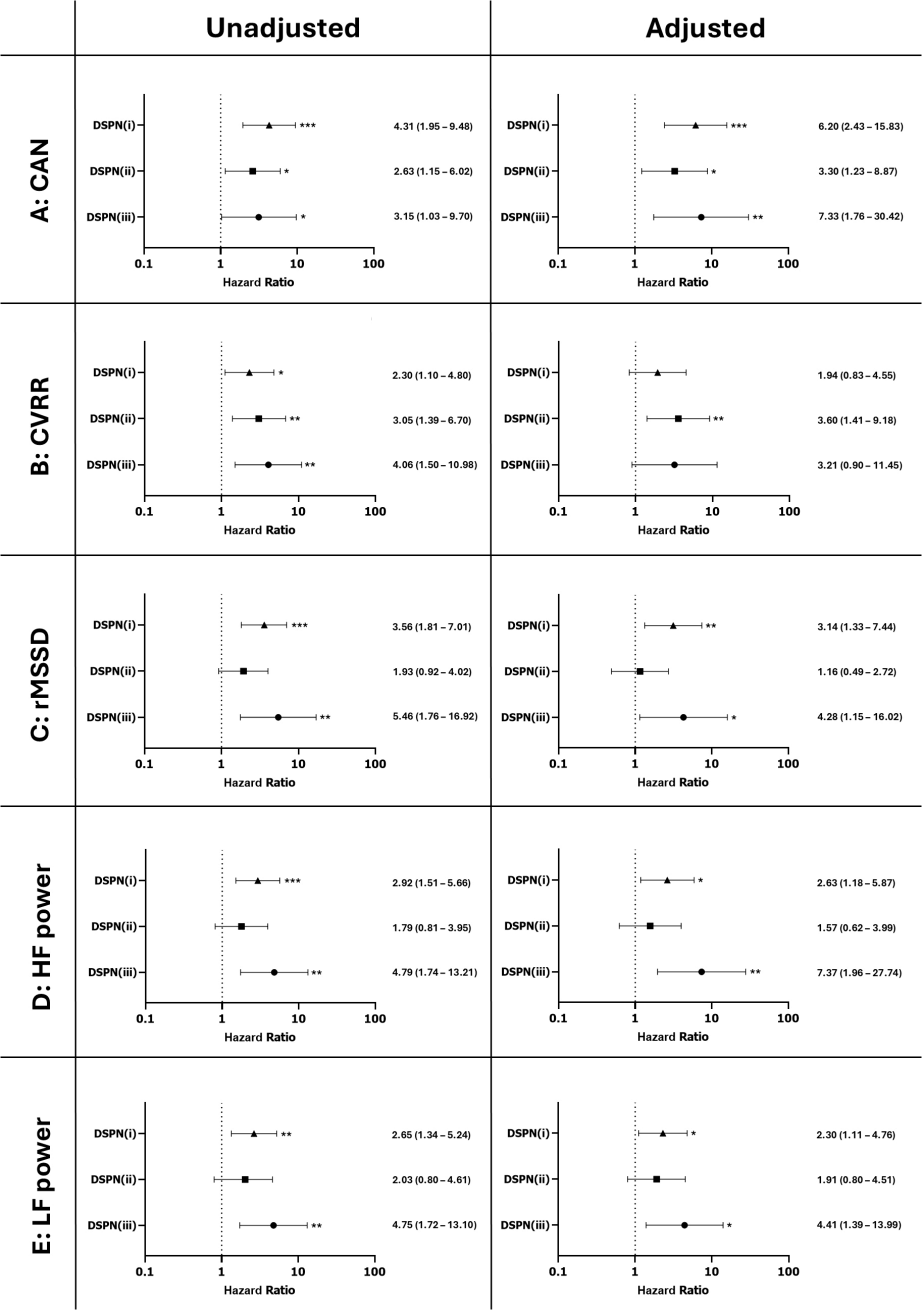
Associations of baseline CAN **(A)** and low HRV **(B–E)** with incident DSPN among participants without DSPN at baseline. *Left panels* show crude hazard ratios (HRs) with 95% confidence intervals; *right panels* present HRs adjusted for age, sex, diabetes duration, body mass index, waist-to-hip ratio, beta-blocker use, eGFR, HbA1c, and uACR. Abbreviations: CAN, cardiovascular autonomic neuropathy; CVRR, coefficient of variation of R–R intervals; rMSSD, root mean square of successive differences; HF power, high-frequency power; LF power, low-frequency power. *, **, *** indicate p <0.05, p <0.01, and p <0.001, respectively.

**Figure 3:**
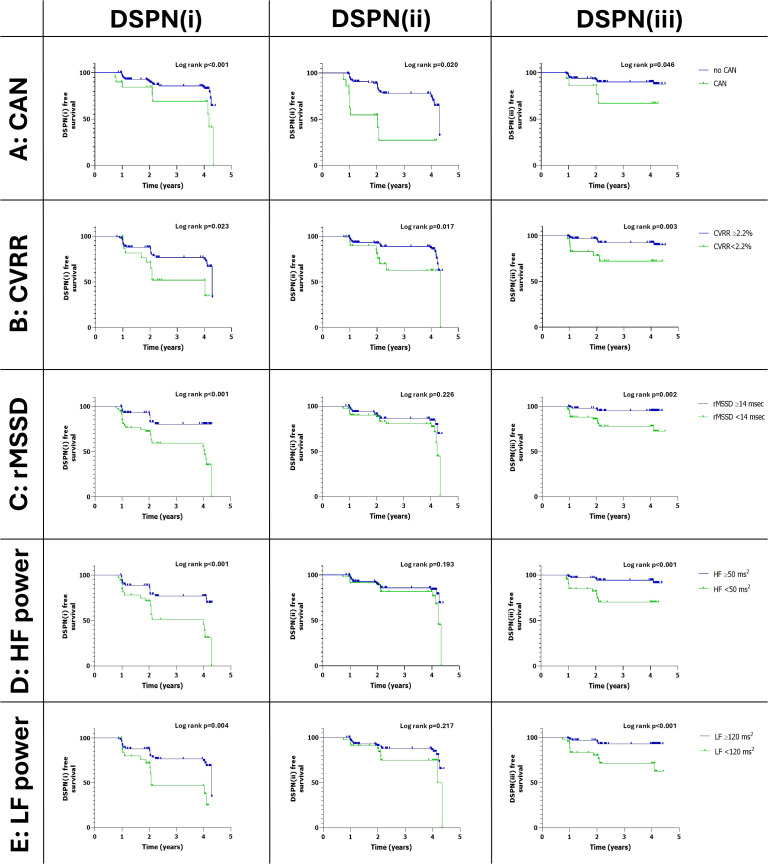
Kaplan–Meier curves for incident DSPN (all three definitions) stratified by baseline CAN or HRV status. Abbreviations: CAN, cardiovascular autonomic neuropathy; CVRR, coefficient of variation of R–R intervals; rMSSD, root mean square of successive differences; HF power, high-frequency power; LF power, low-frequency power.

Baseline CAN was a strong and independent predictor of future DSPN across all definitions, with adjusted hazard ratios of 6.20, 3.30, and 7.33 for DSPN (i), (ii), and (iii), respectively (**Figures 2**, **3**). HRV indices also showed predictive value, although with definition-specific differences. Lower rMSSD, HF power, and LF power independently predicted incident DSPN according to definitions (i) and (iii), whereas for DSPN (ii), only baseline CVRR was significantly associated with future DSPN. These findings indicate that both CAN and reduced HRV are prospectively linked to the development of DSPN, although different HRV metrics appear to capture complementary aspects of risk depending on the DSPN definition applied.

## Discussion

The aim of this study was to determine whether cardiovascular autonomic neuropathy (CAN) and reduced heart rate variability (HRV) predict the development of distal symmetrical polyneuropathy (DSPN) in individuals with diabetes. In a deeply phenotyped cohort assessed using complementary clinical, sensory, and electrophysiological criteria, we found that CAN is not only cross-sectionally associated with DSPN but also represents a strong, independent predictor of incident DSPN over three years. HRV indices showed comparable predictive performance, indicating that autonomic dysfunction—captured through either CARTs or short-term ECG recordings—identifies individuals at elevated neuropathy risk.

Overall, autonomic assessment, either based on CARTs or HRV analysis, may complement clinical and electrophysiological evaluation, aiming to improve risk stratification for DSPN development.

Previous work has consistently documented the high coexistence of CAN and DSPN [6-13], but evidence has been almost exclusively cross-sectional, leaving their temporal relationship unresolved. Our findings address this gap by demonstrating that established CAN predicts future DSPN across three diagnostic definitions. This suggests that autonomic impairment marks a stage of neural vulnerability preceding clinically overt somatic neuropathy. The consistency of predictive associations across clinical, QST-based, and electrophysiological DSPN definitions supports the robustness and generalizability of these observations.

Although causality cannot be inferred, several mechanisms may explain why autonomic dysfunction appears early in the trajectory toward somatic nerve injury. Autonomic fibres are among the most metabolically vulnerable peripheral nerves, and impaired autonomic regulation reflects broader cardiometabolic stress. Reduced HRV has been linked to subtle cardiac remodelling, impaired baroreflex sensitivity, and reduced parasympathetic tone (4, 15–18), features that align with early cardiovascular involvement in metabolic disease. Similar multisystem disturbances are observed in metabolic dysfunction–associated steatotic liver disease (MASLD), where inflammatory activation, lipid imbalance, microvascular rarefaction, and fibrotic remodelling contribute to systemic metabolic instability. These metabolic and vascular perturbations—including endothelial dysfunction, oxidative stress, mitochondrial dysregulation, and renal microangiopathy—are also implicated in DSPN pathogenesis (14, 15, 28). Besides, the presence of CAN has been consistently associated with an increased risk of future cardiovascular events as well as with higher all-cause mortality (29, 30). Thus, CAN and reduced HRV may serve not only as markers of autonomic nerve impairment that precedes the clinical onset of DSPN, but also as integrative indicators of broader multisystem metabolic vulnerability and poorer overall cardiovascular health and prognosis.

Our findings also extend limited prior work on HRV and DSPN. Earlier studies were small and relied on narrow HRV measures and single DSPN definitions. For example, Orlov et al. demonstrated associations between lower HRV and small- and large-fiber measures in individuals with type 1 diabetes (19), although HRV assessment was restricted to R–R interval variability during deep breathing. Another study of individuals with type 2 diabetes found associations between HRV indices and DSPN, although these were partly sex-specific (20), and a third study in elderly participants found higher DSPN prevalence among those with reduced HRV, though DSPN criteria were not well defined (21). Here, a broad set of validated HRV indices showed consistent associations across multiple neuropathy definitions, underscoring their potential clinical utility. Notably, these associations were also observed in individuals without overt clinical DSPN, consistent with autonomic measures capturing early neuropathy-related vulnerability. Given that short resting ECGs are routinely performed in diabetes care, integrating automated HRV analysis into existing ECG workflows would be feasible without additional patient burden or specialised infrastructure. Clinically, abnormal CARTs or low HRV could therefore support earlier identification of individuals who may benefit from closer neuropathy surveillance, structured foot-care follow-up, and intensified optimisation of established risk factors (e.g., glycaemia, adiposity, and albuminuria), with the goal to mitigate downstream complication such as diabetic foot syndrome.

Several methodological considerations should be taken into account when interpreting these findings. The absence of the Valsalva manoeuvre may have reduced the sensitivity of CAN detection and introduced some misclassification; however, the strong and consistent associations observed suggest that any underestimation did not materially affect the results. Previous methodological evaluations have also suggested that E:I and 30:15 responses provide strong diagnostic performance (31, 32), supporting the validity of our approach. Furthermore, the relationship of further HRV metrics, such as the Mean Circular Resultant (MCR), remains to be investigated. MCR is less susceptible to non-respiratory interference, although its availability and clinical validation still remain limited (33). Some variables included in multivariable models may function as mediators rather than confounders, potentially leading to conservative effect estimates. Frequency-domain HRV data were missing in a small subset of participants, and exploratory models yielded unstable odds ratios for WHR and certain HRV parameters; these findings should therefore be viewed as hypothesis-generating. DSPN incidence varied by diagnostic definition, reflecting phenotypic heterogeneity, although associations remained directionally consistent.

In conclusion, this study demonstrates that established CAN is an independent predictor of future DSPN and highlights reduced HRV as a practical alternative marker for identifying individuals at elevated neuropathy risk. These findings support broader implementation of autonomic assessments to improve DSPN risk stratification in clinical practice.

## Author‘s note

Parts of this study were presented in poster form at the EASD Annual Meeting 2025, Vienna, Austria, 15–19 September 2025 and at the Annual Meeting of the German Diabetes Association, Berlin, 28–31 May 2025.

## Data Availability

The raw data supporting the conclusions of this article will be made available by the authors, without undue reservation.
