# Supplementary material for: Reduced heart rate variability predicts incident diabetic polyneuropathy
Source: Front Endocrinol (Lausanne). 2026 Jul 16;17:1880532. doi: 10.3389/fendo.2026.1880532 (PMC13421418; doi:10.3389/fendo.2026.1880532)
Supplement: Supplementary file 1 [file DataSheet1.docx]

|  | Total cohort (n=288) | No CAN (n=241) | CAN (n=47) |
| --- | --- | --- | --- |
| No CART abnormalities | 152 (52.8) | 152 (63.1) | - |
| 1 abnormal CART | 89 (30.9) | 89 (36.9) | - |
| 2 abnormal CARTs | 44 (15.3) | - | 44 (93.6) |
| 3 abnormal CARTs | 3 (1.0) | - | 3 (6.4) |
| Abnormal E/I | 102 (36.6) | 57 (23.7) | 45 (95.7) |
| Abnormal 30/15 | 70 (25.1) | 23 (9.5) | 47 (100.0) |
| Postural Hypotension | 14 (5.0) | 9 (3.9) | 5 (10.6) |

**Supplementary table 1:** Frequencies of CART abnormalities within the whole study sample, as well as among those with and without CAN

| CAN | Unadjusted | | | Adjusted | | |
| --- | --- | --- | --- | --- | --- | --- |
| CVRR | 6.40 | 3.10-13.21 | **<0.001** | 9.94 | 4.14-23.89 | **<0.001** |
| rMSSD | 7.19 | 3.57-14.49 | **<0.001** | 7.29 | 3.39-15.66 | **<0.001** |
| HF power | 5.47 | 2.77-10.82 | **<0.001** | 6.50 | 3.02-14.02 | **<0.001** |
| LF power | 6.21 | 3.14-12.29 | **<0.001** | 8.65 | 3.84-19.50 | **<0.001** |

**Supplementary table 2:** Cross-sectional associations between HRV parameters with the presence of CAN at baseline. Values are (adjusted) ORs, 95% c.i. (for the presented results the following cutoffs were used: CVRR<2.0%, rMSSD<14 msec, HF power <45 ms^2^, LF power <100 ms^2^. Similarly robust associations were present for a range of cutoffs for each parameter, CCRR 2-2.2% rMSSD 12-14 ms, HF 45-50 ms^2^ LF 100-120 ms^2^)

|  | **HbA1c** | **BMI** | **Age** | **Sex (m)** | **ACR** | **DM duration** | **WHR** |
| --- | --- | --- | --- | --- | --- | --- | --- |
| **CAN** | 1.31 (1.05-1.64), p=0.016 | 1.05 (1.00-1.11), p=0.039 | n.s. | n.s. | n.s. | n.s. | n.s. |
| **CVRR<2%** | n.s. | n.s. | 1.06 (1.02-1.10), p=0.001 | n.s. | 1.61 (0.97-2.65), *p=0.065* | n.s. | n.s. |
| **rMSSD<12 msec** | 1.41, (1.15-1.72), p<0.001 | n.s. | n.s. | 1.90 (1.11-3.25), p=0.020 | 1.61 (1.04-2.50), p=0.032 | n.s. | n.s. |
| **HF power <45 ms^2^** | n.s. | n.s. | n.s. | n.s. | 1.54 (0.99-2.41), *p=0.057* | 1.61 (1.12-2.42 ), p=0.010 | 42.04 (1.86-949.0), p=0.019 |
| **LF power<100 ms^2^** | 1.37, (1.10-1.70), p=0.005 | n.s. | 1.04 (1.01-1.06), p=0.012 | n.s. | n.s. | n.s. | 39.78 (1.10-1582.3), p=0.049 |
| **DSPN (i)** | n.s. | n.s. | 1.07 (1.04-1.10), p<0.001 | n.s. | 1.48 (0.95-2.29), *p=0.083* | n.s. | n.s. |
| **DSPN (ii)** | n.s. | n.s. | 1.05 (1.02-1.08), p=0.004 | n.s. | 2.42 (1.47-3.97),  p<0.001 | n.s. | n.s. |
| **DSPN (iii)** | n.s. | n.s. | 1.07 (1.03-1.10), p<0.001 | 6.62 (2.76-15.87), p<0.001 | 2.34 (1.36-4.02),  p=0.002 | n.s. | n.s. |

**Supplementary table 3:**. Factors associated with the presence of CAN or low HRV on baseline. Presented values are adjusted Odds Ratios (95% c.i) derived from multivariable binary logistic regression. Among tested parameters, eGFR and beta blocker use were not associated with any of the dependent variables. ACR and DM duration were log transformed for the analysis.


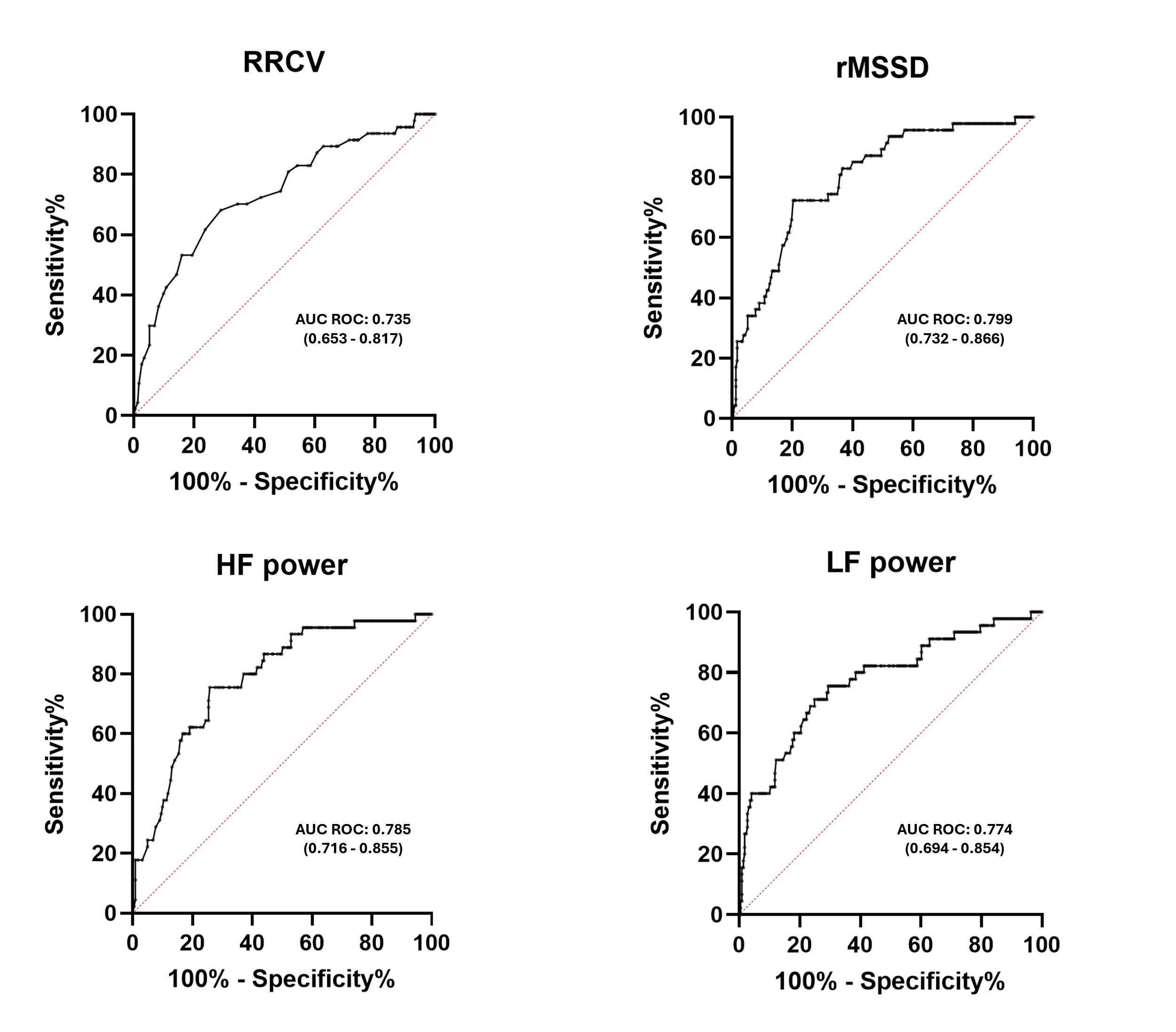


**Supplementary figure 1:** ROC curves for the performance of common heart rate variability indices for predicting cardiac autonomic neuropathy, diagnosed based on cardiovascular autonomic reflex tests. The following Youden (J) indexes apply for the cutoffs chosen for the analysis: RRCV<2%: Sensitivity 42.5%, Specificity 89.2%, J-index 0.32 <2.2%: Sensitivity 53.2%, Specificity 84.1%, J-index 0.37, rMSSD <12 msec: Sensitivity 72.3%, Specificity 74.1%, J-index 0.46 <14 msec: Sensitivity 83.0%, Specificity 63.4%, J-index 0.46 , HF-power <45 ms2: Sensitivity 64.4%, Specificity 75.6%, J-index 0.40, <50 ms2: Sensitivity 75.6%, Specificity 72.4%, J-index 0.38, LF-power <100 ms2: Sensitivity 60.0%, Specificity 80.5%, J-index 0.41, <120 ms2: Sensitivity 71.1%, Specificity 75.1%, J-index 0.36.

**Establishment of normal values for lower extremity nerve conduction studies**

Percentiles of sensory (Sural) and motor (Common Peroneal, Tibial) nerve conduction velocities and action potentials derived from a group of 161 individuals without DM (age 52.4±12.7 years, 59.0% women) participating in HEIST-DiC, without neuropathic symptoms or deficits (Neuropathy Symptoms Score and Neuropathy Disability Score ≤ 2). Cut off values were as follows: Sural nerve conduction velocity <38.7 m/sec (1^st^ percentile), Sural Nerve Action Potential <2.53 μV (1^st^ percentile), Common Peroneal conduction velocity <37.0 m/sec (2.5^st^ percentile), Common Peroneal compound muscle action potential <1.14 μV (2.5^st^ percentile), Tibial conduction velocity <38.0 m/sec (2.5^st^ percentile), Tibial compound muscle action potential <2.72 μV (2.5^st^ percentile).
